# Supplementary material for: Sensing the ortho Positions in C6Cl6 and C6H4Cl2 from Cl2− Formation upon Molecular Reduction
Source: Molecules. 2022 Jul 27;27(15):4820. doi: 10.3390/molecules27154820 (PMC9369944; doi:10.3390/molecules27154820)
Supplement: Supplementary file 1 [file molecules-27-04820-s001.zip › molecules-1825676-supplementary.pdf]

Supplementary Materials

Sensing the *ortho* Positions in C_6Cl_6 and $C_6H_4Cl_2$ from Cl_2^- Formation upon Molecular Reduction

Sarvesh Kumar [1], José Romero [1,2], Michael Probst [2,3], Thana Maihom [4], Gustavo García [5] and Paulo Limão-Vieira [1,*]

1. Atomic and Molecular Collisions Laboratory, CEFITEC, Department of Physics, Universidade NOVA de Lisboa, Campus de Caparica, 2829-516 Caparica, Portugal; s.kumar@campus.fct.unl.pt
2. Institut für Ionenphysik und Angewandte Physik, Leopold-Franzens Universität Innsbruck, Technikerstraße 25, 6020 Innsbruck, Austria; j.romero@campus.fct.unl.pt
3. School of Molecular Science and Engineering, Vidyasirimedhi Institute of Science and Technology Rayong 21210, Thailand; Michael.Probst@uibk.ac.at
4. Department of Chemistry, Faculty of Liberal Arts and Science, Kasetsart University, Kamphaeng Saen Campus, Nakhon Pathom 73140, Thailand; t_maihom@hotmail.com
5. Instituto de Física Fundamental, Consejo Superior de Investigaciones Científicas, Serrano 113-bis, 28006 Madrid, Spain; g.garcia@csic.es

* Correspondence: Michael.Probst@uibk.ac.at (M.P.); plimaovieira@fct.unl.pt (P.L.-V.);

Figure captions

Figure S1: Time-of-flight mass spectra of negative ions formed in electron transfer experiments from potassium atoms to hexachlorobenzene (C_6Cl_6) and dichlorobenzene (DCB) isomers at 100 eV collision energy in the lab frame.

Figure S2: Energy profile for the detachment of a Cl_2 (Cl_2^-) fragment if the Cl_2 (Cl_2^-) distance is frozen at the equilibrium value of the isolated diatomic.

Table captions

Table S1: Thresholds for the dissociation of Cl_2 and Cl_2^- from neutral and anionic C_6Cl_6 calculated with three model chemistries. Energies are given in eV.

Table S2: Thresholds for the dissociation of Cl_2 and Cl_2^- from $C_6Cl_2H_4$ calculated with various model chemistries. Energies are given in eV.

Table S3: Adiabatic electron affinities of C_6Cl_6, C_6Cl_4 and Cl_2 from various model chemistries.

Table S4: Vibrational modes of neutral C_6Cl_6 and its anion, indicating out-of-plane modes (B3LYP-GD3/aug-cc-pVTZ calculations).

Table S5: Vibrational modes of all three monomers of $C_6H_4Cl_2$ neutral molecules, from B3LYP-GD3/aug-cc-pVTZ calculations.

(1) Time-of-flight mass spectra of negative ions from C_6Cl_6 and $C_6H_4Cl_2$ isomers

In the Cl_2^- TOF mass contribution, the peak should show its isotope contributions at 70 u (100%), 72 u (~65%), and 74 u (~11%). A close inspection of this anion feature in Figure S3 reveals that the peak contains three contributions but not in the expected intensity. Given that the limited TOF mass resolution (m/Δm ≈ 125) does not allow clearly resolving close fragment anions, yet a proper peak fitting, reproduces perfectly well the expected isotope distribution intensities.

Figure S1: Time-of-flight mass spectra of negative ions formed in electron transfer experiments from potassium atoms to hexachlorobenzene (C_6Cl_6) and dichlorobenzene (DCB) isomers at 100 eV collision energy in the lab frame.

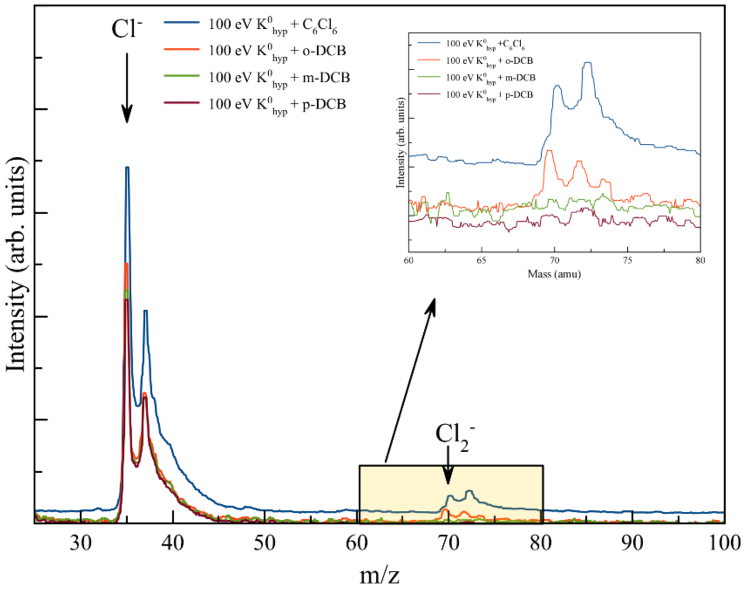

(2) Symmetric detachment of Cl_2^- from $C_6Cl_6^-$

The hypothetical symmetric direct detachment of Cl_2 was investigated by calculating relaxed energy profiles with the distance between the midpoint of two *ortho* Cl atoms and the center of the C_6Cl_4 unit is plotted as reaction coordinate. Cl-Cl and C-C bonds are fixed to their equilibrium bond lengths in the dissociated fragments while all other internal degrees of freedom are relaxed at each point. The energies are plotted relative to C_6Cl_6 ($C_6Cl_6^-$) at their equilibrium geometries.

Figure S2: Reaction coordinate for the detachment of a Cl_2 fragment. See text for explanations.

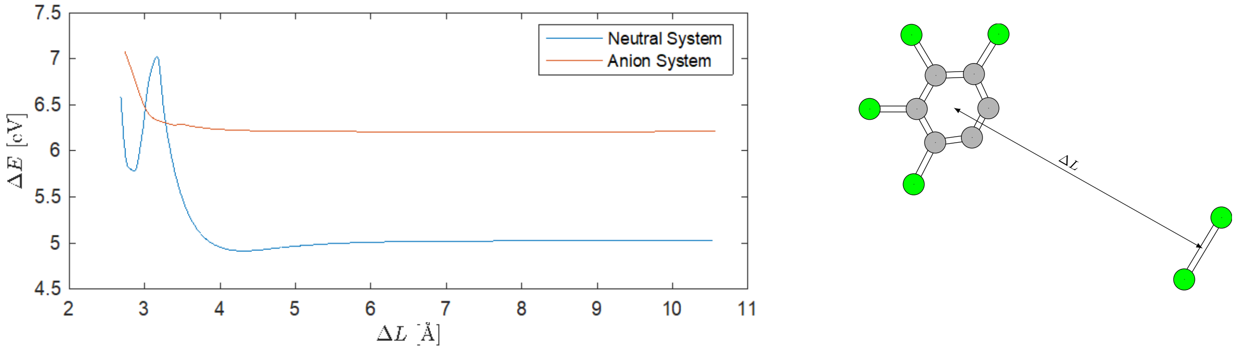

(3) Dissociation thresholds of Cl_2 and Cl_2^- and adiabatic electron affinities

Dissociation thresholds of Cl_2 and Cl_2^- fragments were calculated from neutral and anionic C_6Cl_6 for the *ortho*, *meta* and *para* monomers by subtracting the sum of the energies of the products and the energy of the molecule before fragmentation. The dissociation thresholds are calculated with the quantum thermochemical extrapolation methods G4MP2 and CBS-QB3. Energies from B3LYP-GD3/aug-cc-pVTZ calculations, also obtained from fully optimized geometries are also included.

Table S1: Thresholds for the dissociation of Cl_2 and Cl_2^- from neutral and anionic C_6Cl_6 calculated with three model chemistries. Energies are given in eV.

			B3LYP-GD3 aug-cc-pVTZ	G4MP2	CBS-QB3
C_6Cl_6	$\longrightarrow$	$C_6Cl_4\ (ortho) + Cl_2$	4.12	4.31	4.47
	$\longrightarrow$	$C_6Cl_4\ (meta) + Cl_2$	4.31	4.75	4.89
	$\longrightarrow$	$C_6Cl_4\ (para) + Cl_2$	5.64	6.01	6.61
$C_6Cl_6^-$	$\longrightarrow$	$C_6Cl_4\ (ortho) + Cl_2^-$	2.52	2.96	2.87
	$\longrightarrow$	$C_6Cl_4\ (meta) + Cl_2^-$	2.70	3.40	3.29
	$\longrightarrow$	$C_6Cl_4\ (para) + Cl_2^-$	4.04	4.56	4.56
	$\longrightarrow$	$C_6Cl_4^-\ (ortho) + Cl_2$	3.01	3.14	3.27
	$\longrightarrow$	$C_6Cl_4^-\ (meta) + Cl_2$	3.02	3.40	3.41
	$\longrightarrow$	$C_6Cl_4^-\ (para) + Cl_2$	3.39	3.89	3.79
$C_6Cl_6 + e^-$	$\longrightarrow$	$C_6Cl_4\ (ortho) + Cl_2^-$	1.25	1.96	1.97
	$\longrightarrow$	$C_6Cl_4\ (meta) + Cl_2^-$	1.43	2.39	2.39
	$\longrightarrow$	$C_6Cl_4\ (para) + Cl_2^-$	2.77	3.65	3.66
	$\longrightarrow$	$C_6Cl_4^-\ (ortho) + Cl_2$	1.74	2.14	2.38
	$\longrightarrow$	$C_6Cl_4^-\ (meta) + Cl_2$	1.75	2.39	2.52
	$\longrightarrow$	$C_6Cl_4^-\ (para) + Cl_2$	2.11	2.88	2.89

Table S2: Thresholds for the dissociation of Cl_2 and Cl_2^- from $C_6Cl_2H_4$ calculated with various model chemistries. Energies are given in eV.

			B3LYP-GD3 aug-cc-pVTZ [eV]	G4MP2 [eV]	CBS-QB3 [eV]
$C_6Cl_2H_4(ortho)$	$\longrightarrow$	$C_6H_4\ (ortho) + Cl_2$	4.40	4.42	4.58
$C_6Cl_2H_4^-(ortho)$	$\longrightarrow$	$C_6H_4\ (ortho) + Cl_2^-$	1.39	1.95	1.88
	$\longrightarrow$	$C_6H_4^-\ (ortho) + Cl_2$	3.42	3.68	3.79
$C_6Cl_2H_4(ortho) + e^-$	$\longrightarrow$	$C_6H_4\ (ortho) + Cl_2^-$	1.53	2.07	2.08
	$\longrightarrow$	$C_6H_4^-\ (ortho) + Cl_2$	3.56	3.80	3.99
$C_6Cl_2H_4(meta)$	$\longrightarrow$	$C_6H_4\ (meta) + Cl_2$	5.04	5.15	5.27
$C_6Cl_2H_4^-(meta)$	$\longrightarrow$	$C_6H_4\ (meta) + Cl_2^-$	1.97	2.68	2.57
	$\longrightarrow$	$C_6H_4^-\ (meta) + Cl_2$	3.73	4.10	4.19
$C_6Cl_2H_4(meta) + e^-$	$\longrightarrow$	$C_6H_4\ (meta) + Cl_2^-$	2.16	2.79	2.78
	$\longrightarrow$	$C_6H_4^-\ (meta) + Cl_2$	3.92	4.21	4.40
$C_6Cl_2H_4(para)$	$\longrightarrow$	$C_6H_4\ (para) + Cl_2$	6.11	5.65	5.79
$C_6Cl_2H_4^-(para)$	$\longrightarrow$	$C_6H_4\ (para) + Cl_2^-$	3.06	2.79	2.78
	$\longrightarrow$	$C_6H_4^-\ (para) + Cl_2$	3.95	4.21	4.05
$C_6Cl_2H_4(para) + e^-$	$\longrightarrow$	$C_6H_4\ (para) + Cl_2^-$	3.23	3.29	3.29
	$\longrightarrow$	$C_6H_4^-\ (para) + Cl_2$	4.12	4.50	4.48

(4) Adiabatic electron affinities of $C_6Cl_2H_4$ and related fragments from dissociation

Table S3: Adiabatic electron affinities of C_6Cl_6, C_6Cl_4 and Cl_2 from various model chemistries.

	B3LYP-GD3 aug-cc-pVTZ [eV]	G4MP2 [eV]	CBS-QB3 [eV]
C_6Cl_6	1.27	1.01	0.89
$C_6Cl_4(ortho)$	2.38	2.18	2.09
$C_6Cl_4(meta)$	2.56	2.36	2.37
$C_6Cl_4(para)$	3.53	3.13	3.27
Cl_2	2.88	2.36	2.50

Vibrational modes of C_6Cl_6 and $C_6H_4Cl_2$ monomers

Symmetry breaking via an out-of-plane (oop) vibration can trigger a detachment reaction by allowing transfer of an electron from a metastable but potentially long-lived state to an antibonding sigma orbital. Table S4 distinguishes between oop and other vibrations of C_6Cl_6. All lowest modes have oop character. The same is true for all $C_6H_4Cl_2$ isomers (Table S5). The very low oop frequencies present indicate that an electron captured in a dipolar or π orbital can easily move on to potentially weaken a C-Cl bond.

Table S4: Vibrational modes of neutral C_6Cl_6 and its anion, indicating out-of-plane modes (B3LYP-GD3/aug-cc-pVTZ calculations).

C_6Cl_6		$C_6Cl_6^-$	
Frquency [cm^{-1}]	oop mode ?	Frquency [cm^{-1}]	oop mode ?
64,15	Yes	10,18	Yes
64,22	Yes	57,63	Yes
89,61	Yes	61,74	Yes
166,91	Yes	130,62	Yes
216,82	No	180,64	No
216,87	No	187,62	No
221,82	No	189,77	No
221,83	No	197,96	No
237,95	No	208,54	No
320,99	No	285,97	No
321,01	No	286,14	No
340,96	Yes	293,23	Yes
340,97	Yes	310,34	Yes
367,20	No	322,88	No
389,37	No	342,02	Yes
615,86	Yes	487,79	No
615,89	Yes	492,28	No
625,56	No	530,56	Yes
683,90	No	537,87	Yes
683,95	No	614,83	No
733,55	Yes	681,68	Yes
871,36	No	703,58	No
871,51	No	704,56	No
1086,80	No	931,54	No
1207,32	No	1041,10	No
1241,23	No	1219,75	No
1344,61	No	1243,65	No
1344,67	No	1377,33	No
1527,63	No	1444,18	No
1527,68	No	1535,65	No

Table S5: Vibrational modes of all three monomers of $C_6H_4Cl_2$ neutral molecules, from B3LYP-GD3/aug-cc-pVTZ calculations.

ortho		para		meta	
Frquency [cm^{-1}]	oop mode ?	Frquency [cm^{-1}]	oop mode ?	Frquency [cm^{-1}]	oop mode ?
134,79	Yes	100,79	Yes	166,21	Yes
200,12	No	221,68	No	198,28	No
233,36	Yes	295,18	Yes	202,09	Yes
340,65	No	329,04	No	371,65	No
426,70	No	359,88	No	398,25	No
450,48	Yes	418,21	Yes	432,07	No
477,51	No	497,72	Yes	443,82	Yes
524,54	Yes	540,86	No	548,99	Yes
670,76	No	640,24	No	674,06	No
709,14	Yes	710,12	Yes	692,99	Yes
747,15	No	754,29	No	783,85	No
769,31	Yes	834,37	Yes	796,38	Yes
878,59	Yes	846,18	Yes	901,27	Yes
971,58	Yes	967,15	Yes	920,62	Yes
1002,40	Yes	982,74	Yes	1000,16	Yes
1045,59	No	1032,10	No	1016,56	No
1060,13	No	1100,12	No	1098,84	No
1141,26	No	1103,50	No	1105,77	No
1154,48	No	1134,68	No	1133,75	No
1194,70	No	1207,86	No	1199,12	No
1286,42	No	1301,19	No	1292,58	No
1304,04	No	1326,25	No	1334,81	No
1464,81	No	1424,40	No	1444,06	No
1492,89	No	1509,84	No	1496,62	No
1605,57	No	1608,70	No	1609,67	No
1614,30	No	1613,24	No	1611,12	No
3176,31	No	3193,57	No	3178,38	No
3189,32	No	3194,87	No	3204,63	No
3200,22	No	3207,50	No	3208,95	No
3204,87	No	3209,13	No	3214,22	No
